# A case of vascular Ehlers–Danlos syndrome with a ruptured hepatic artery after surgical treatment of peritonitis caused by the perforation of the colon

**DOI:** 10.1186/s40792-021-01156-0

**Published:** 2021-03-23

**Authors:** Daisuke Kakinuma, Takeshi Yamada, Yoshikazu Kanazawa, Kunihiko Matsuno, Tomoko Sahara, Hiroshi Yoshida

**Affiliations:** 1 Department of Surgery, Nippon Medical School Chiba Hokusoh Hospital, 1715 Kamagari, Inzai, Chiba 270-1694 Japan; 2 Department of Gastrointestinal and Hepato-Biliary-Pancreatic Surgery, Nippon Medical School, 1-1-5, Sendagi, Bunkyo-ku, Tokyo, 113-8603 Japan; 3 Division of Personalized Genetic Medicine, Nippon Medical School, 1-1-5, Sendagi, Bunkyo-ku, Tokyo, 113-8603 Japan

**Keywords:** Vascular type of Ehlers–Danlos syndrome, Sigmoid colon perforation, Hepatic artery rupture, Pan-peritonitis, Remote vascular catastrophe

## Abstract

**Background:**

Ehlers–Danlos syndrome (EDS) is an inherited disorder that causes connective tissue fragility. The vascular type of EDS (vEDS) caused by defective collagen type III production accounts for 5%–10% of all EDS cases. Patients can develop gastrointestinal or arterial ruptures, which cause poor prognosis. We report a case of a patient who experienced colonic rupture, which was immediately followed by arterial rupture.

**Case presentation:**

A 40-year-old man who had been genetically diagnosed with vEDS 6 years previously was admitted to our hospital with ischemic colitis. After 3 days of conservative treatment, his abdominal pain worsened, and computed tomography (CT) revealed free air in the abdominal cavity. Pan-peritonitis due to perforation of the sigmoid colon was diagnosed. Intraperitoneal lavage and drainage and Hartmann’s operation were urgently performed. Because the patient had confirmed vEDS, we performed the surgery in a protective manner. The postoperative course was initially good, and he was transferred to the general ward 3 days after surgery. However, 5 days after surgery, massive intra-abdominal hemorrhage suddenly occurred, and contrast-enhanced CT showed an aneurysm in the common hepatic artery that had ruptured; this aneurysm was not present before surgery and was far from the surgical field. Although we considered an emergency operation, the patient suddenly experienced cardiac arrest and was unresponsive to resuscitation.

**Conclusions:**

In cases of vEDS, vascular rupture can occur immediately after surgery for intestinal rupture. We recommend paying special attention to vascular complications in patients in their forties, as such complications are the most common causes of death.

## Background

Ehlers–Danlos syndrome (EDS) is an autosomal dominant inherited disease in which abnormal collagen metabolism causes systemic connective tissue fragility in the skin, joints, blood vessels, and other structures. There are six distinct types of EDS: classical, hypermobile, vascular, kyphoscoliosis, arthrochalasia, and dermatosparaxis [1]. These differ in severity, are associated with different organs, and affect patient well-being by inducing various clinical symptoms. Among them, the vascular type of EDS (vEDS), which accounts for 5%–10% of all EDS cases [2], has the worst prognosis owing to its high potential for arterial rupture and gastrointestinal perforation; its estimated prevalence is 1:50,000 [3]. It is caused by a mutation in *COL3A1,* which affects type III procollagen synthesis [4–6].

*COL3A1* is the only gene implicated in vEDS to date. In a typical vEDS case, its mutation results in abnormal production of type III procollagen and consequent tissue fragility, as well as arterial rupture and dissection, gastrointestinal perforation, uterine rupture in women, and other characteristic features. We report a case of a man with confirmed vEDS who died from a hepatic artery rupture triggered by a 2-cm perforation of the sigmoid colon.

## Case presentation

A 40-year-old man was admitted to our hospital with a chief complaint of lower abdominal pain and melena. His medical history included surgery for acute brachiocephalic dissection at the age of 17 years. He was diagnosed with vEDS by genetic testing, which found missense variants in *COL3A1*, while hospitalized for symptoms associated with hemothorax at the age of 34. He had no family history of vEDS; however, his mother had died suddenly due to acute aortic dissection.

**Fig. 1:**
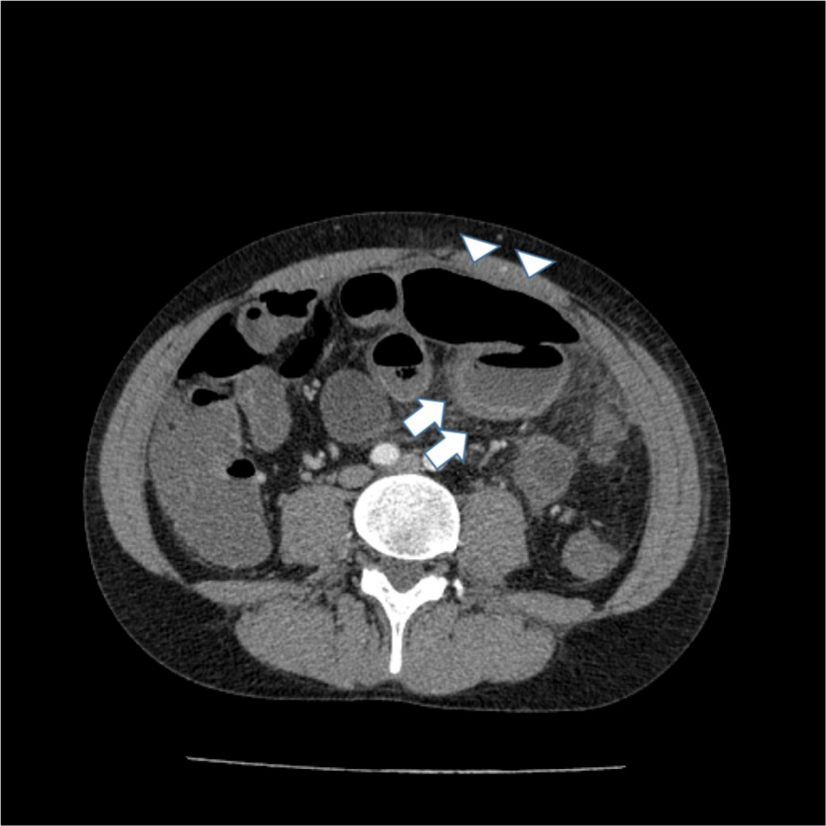
Abdominal computed tomography findings on the third day after patient admission to the hospital for abdominal pain. Wall thickening and thinning of the sigmoid colon (white arrows) and free gas in the abdominal cavity (white triangles) are observed

**Fig. 2:**
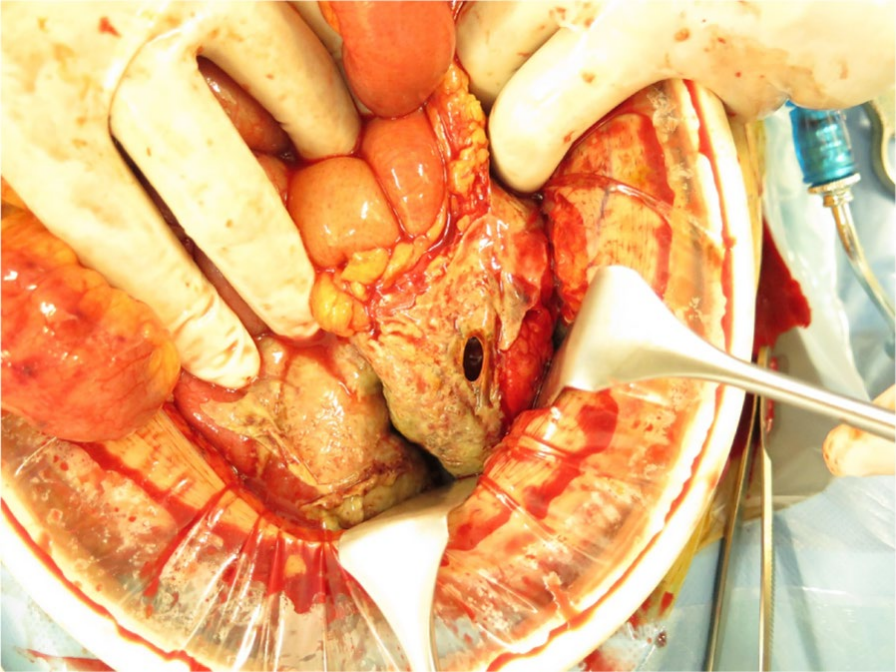
Intraoperative findings. A 2-cm perforation is found in the sigmoid colon

**Fig. 3:**
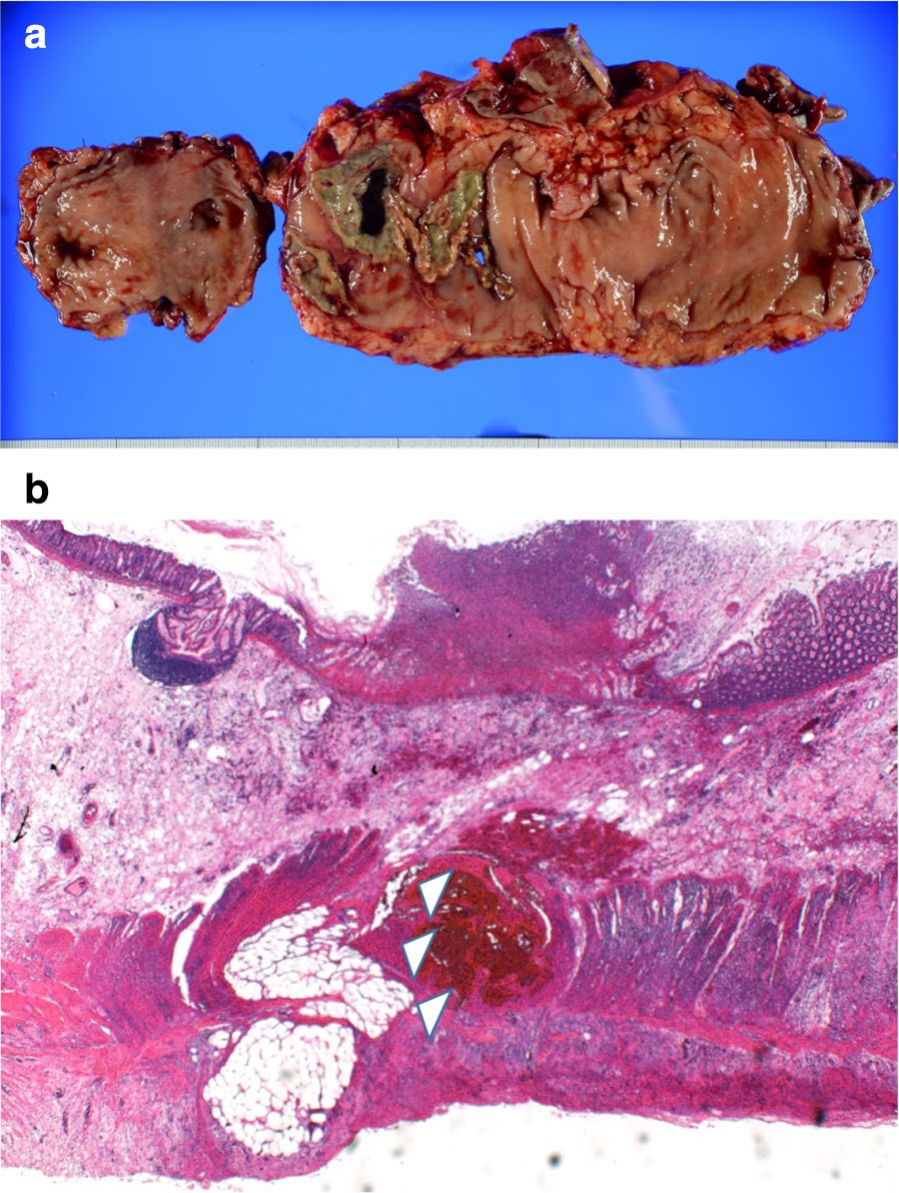
**a** Perforated sigmoid colon. **b** Infiltration of inflammatory cells and rupture of the muscular layer (white triangles) are observed, and collagen fibers in the submucosal layer tend to be slightly thin

At our hospital, he was diagnosed with ischemic colitis and conservatively treated by fasting and with antibiotics. Three days after hospital admission, his symptoms exacerbated, and abdominal computed tomography (CT) showed intraperitoneal free air and thickening and thinning of the sigmoid colon wall (Fig. 1). Pan-peritonitis caused by the ischemic colitis and consequent perforation of the sigmoid colon was diagnosed. An emergency operation was performed, and contamination of the abdominal cavity with feces was intraoperatively revealed. Moreover, the colonic wall was thinned and edematous, the tissue color was poor from the descending colon to the sigmoid colon, and a 2-cm perforation was observed in the sigmoid colon (Fig. 2). As we expected, the tissue was very fragile and bled easily; hence, we performed the surgery more protectively than usual. We resected the descending colon and sigmoid colon, washed the abdominal cavity with 10 L of saline, and constructed a colostomy in the transverse colon. We confirmed the upper abdominal cavity during intraperitoneal lavage, but no obvious organ damage including common hepatic artery was observed. We confirmed sufficient hemostasis and placed two prophylactic drain tubes at the rectovesical pouch and left subdiaphragm. Macroscopically, a perforation was found in the sigmoid colon (Fig. 3a). Microscopically, infiltration of inflammatory cells and rupture of the muscular layer were observed, and collagen fibers in the submucosal layer tended to be slightly thin (Fig. 3b).

**Fig. 4:**
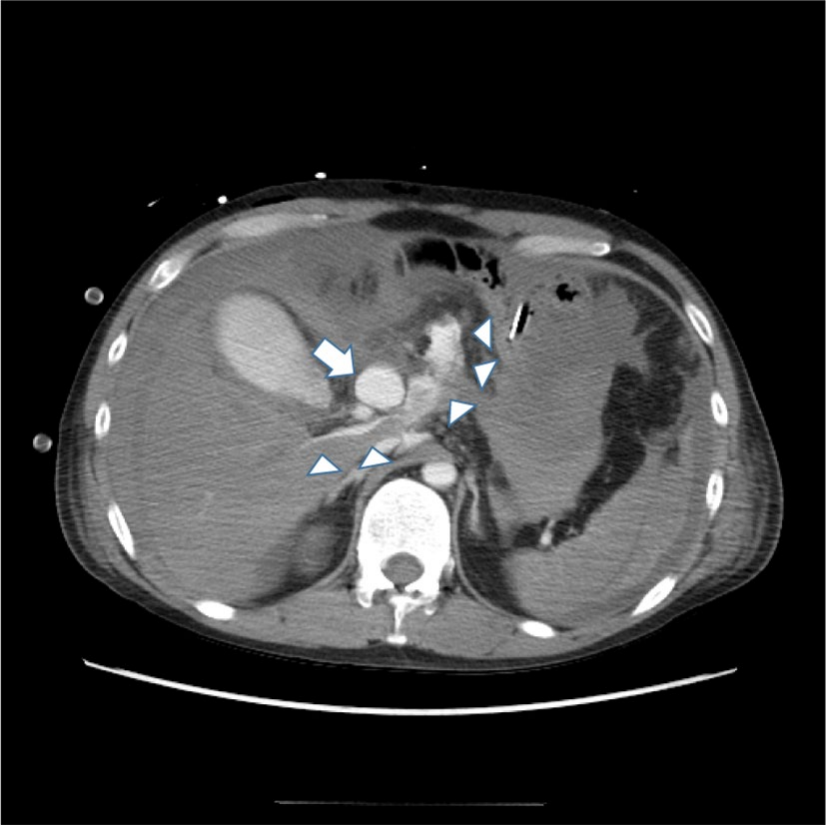
Abdominal computed tomography findings on the fifth day after surgical sigmoid colon resection and colostomy construction. An aneurysm in the common hepatic artery (white arrow) and extravasation (white triangles) are observed

After the operation, the patient entered the intensive care unit and was managed systemically. Because there were no initial drain-related problems, he was moved to the general ward 3 days after surgery. Five days after surgery, bleeding from the abdominal drainage tube suddenly occurred, and the patient had low blood pressure and was unconscious. Enhanced CT revealed a common hepatic aneurysm rupture that was not present before the surgery (Fig. 4). We considered emergency surgery or intravascular treatment, but the patient went into cardiopulmonary arrest, did not respond to resuscitation, and died.

## Discussion

The most significant point in this case is that the vascular ruptured occurred immediately after surgery for intestinal rupture. Intestinal and vascular ruptures are common complications in patients with vEDS. In fact, Pepin et al. reported that 15% of patients with vEDS with intestinal complications had vascular complications during follow-up [3], and Watanabe et al. also reported that 66% of Japanese patients with vEDS have intestinal and vascular complications [7]. However, to the best of our knowledge, this is the first report of vEDS in a patient who experienced intestinal complications, which was immediately followed by vascular complications. In this case, no aneurysm was found on preoperative contrast-enhanced abdominal CT, indicating that gastrointestinal surgery for pan-peritonitis triggered the vascular complication. It is well known that vascular complications can develop after endovascular treatment [8–10]. Horowitz et al. observed splenic artery and cardiac ruptures with unknown causes 3 and 21 days after endovascular treatment, respectively, and referred these events as “remote vascular catastrophes” [11]. The exact cause of the catastrophes is unknown, but elevated collagenase activity after invasive treatment may be a potential culprit [12]. In this case, two major invasions, surgical colon resection and pan-peritonitis, were added to the patient; thus, it is possible that there was a potential increase in collagenase activity. In addition, it cannot be denied that light traction or exclusion was applied to the upper abdominal cavity around common hepatic artery, but finally, we confirmed that there was no obvious organ damage. Thus, the vascular complication in our patient may be a remote vascular catastrophe caused by surgery for pan-peritonitis. He had no aneurysm before surgery, so performing contrast-enhanced CT after surgery might help detect such remote vascular catastrophes.

We should pay special attention to vascular complications in patients in their forties. The average age of death from vEDS is 48 years [3]. Intestinal rupture is a serious complication in patients with vEDS, but is rarely fatal with proper treatment [3]. In fact, most causes of death in patients with vEDS are related to vascular rupture, and bowel rupture accounts for 8% of all vEDS patient deaths [3]. Surgical mortality is reported to be 7% [13]. Vascular complication is often fatal. Thus, given the average life expectancy of patients with vEDS, we should pay attention to vascular complications in patients. Our patient had taken celiprolol (400 mg/day) before the surgery, because celiprolol reduces vascular complications in patients with vEDS [14]. However, he was not administered celiprolol after the surgery. Immediately after surgery, systolic blood pressure was as low as 80–90 mmHg due to sepsis. However, the general condition improved; he started to get out of bed, and the systolic blood pressure often increased to 130 mmHg due to wound pain. Blood pressure monitoring was already discontinued in the general ward, and blood pressure may have risen further. Although there were no reports showing perioperative blood pressure control goals, rapid changes in blood pressure under general anesthesia have been reported to promote bleeding [15]. In addition, cases of arterial rupture due to a temporary increase in blood pressure during labor have also been reported [3]. Since intestinal perforation is an emergency operation, epidural anesthesia is often not indwelled. Good pain control and continued blood pressure monitoring will be important when starting to get out of bed. Furthermore, it is unclear whether 6-day cessation of the drug can trigger vascular complications, so immediate resurgence after surgery may be ideal.

Constipation treatment is important for patients with vEDS. Although the direct cause of intestinal perforation in this case is ischemic colitis, chronic constipation can increase intestinal pressure and induce the ischemic colitis. In fact, the patient had been complaining of constipation for some time, but had not been given regular laxatives to relieve the intestinal pressure. Approximately half of patients with vEDS have chronic constipation, which causes intestinal rupture [16, 17]. However, it is unclear whether constipation is the main trigger of colonic perforation, because the majority of first perforations occur before genetic diagnosis [13].

We should never rule out vEDS when there is no family history of vEDS or the characteristic physical (thin, transparent skin that bleeds easily and acrogeria) and facial (thin lips, small jaw, sharp nose, and bulging eyes) features. Although our patient had no family history and no characteristic features, he was diagnosed with vEDS, because he experienced serious vascular complications at a young age. We previously reported 28-year-old [18] and 52-year-old [19] patients with vEDS who had no family history. Only 38% of patients with vEDS have a family history [3]. Skin hyperextension and joint hypermobility are milder in vEDS than in the other EDS types, and many cases of vEDS are not diagnosed until serious symptoms appear [2, 3, 20]. The patient in our case underwent surgery for acute brachiocephalic dissection and hemothorax at a young age, and his mother had died suddenly due to acute aortic dissection. Hence, we strongly suspected him to have vEDS.

At the time of surgery, close attention to the patient is needed as patients with vEDS tend to bleed during surgery [16, 21, 22]. Anastomosis at emergency surgery is a great risk for patients with vEDS [18, 19, 23, 24]. It is also controversial whether colostomy closure should be performed. Many young patients with vEDS want closure of colostomy [18]; however, stoma closures have a great risk of anastomotic leakage. Approximately half of patients who experienced colonic perforation develop re-perforation [13]. However, patients with stoma often experienced re-perforation too. Thus, elective subtotal colectomy after a Hartmann procedure has been advocated by several authors [13].

## Conclusion

In conclusion, vascular rupture, one of the remote vascular catastrophe, can occur immediately after surgery for intestinal rupture. We should pay special attention to patients in their forties as vascular complication is the most common cause of death. We should pay more attention to chronic constipation, which is reported in approximately half of the patients with vEDS, because it may be a cause of intestinal rupture.

## Data Availability

Not applicable.

## Abbreviations

CT: Computed tomography
EDS: Ehlers–Danlos syndrome
vEDS: Vascular type of Ehlers–Danlos syndrome
